# Sodium valproate ameliorates aluminum-induced oxidative stress and apoptosis of PC12 cells

**DOI:** 10.22038/ijbms.2019.36930.8804

**Published:** 2019-11

**Authors:** Forough Iranpak, Jamileh Saberzadeh, Mahmood Vessal, Mohammad Ali Takhshid

**Affiliations:** 1 Department of Biochemistry, Islamic Azad University of Shiraz, Shiraz, Iran; 2 Diagnostic Laboratory Sciences and Technology Research Center, Shiraz University of Medical Sciences, Shiraz, Iran

**Keywords:** Aluminum maltolate, Apoptosis, Histone deacetylase – inhibitor, Oxidative stress, Valproic acid

## Abstract

**Objective(s):**

According to recent studies, valproate shows some protection against oxidative stress (OS) induced by neurotoxins. Current investigation tried to determine the possible ameliorating effects of sodium valproate (SV) against aluminum (Al)-induced cell death, apoptosis, mitochondrial membrane potential (MMP), and OS in PC12 cells.

**Materials and Methods:**

In this in vitro study, PC12 cells were treated with different concentrations of aluminum maltolate (Almal) with and without SV (50–400 µM). Cell viability was assessed by MTT assay. To measure quantitatively the effects of SV on Al-induced apoptosis and reactive oxygen species (ROS), flowcytometry using 7AAD/annexin-V and 2’, 7’-dichlorofluorescein diacetate staining were employed, respectively. MMP was monitored using the retention of rhodamine 123. Catalase (CAT) activity was assayed by the rate of decomposition of hydrogen peroxide.

**Results:**

Exposure of PC12 cells for 48 hr to Almal (125–2000 µM) significantly reduced cell viability (IC_50_=1090 μM), increased ROS generation and apoptosis, and reduced MMP and CAT activity. SV reduced the Almal-induced cell death and apoptosis. Furthermore, the effects of Almal on ROS generation, catalase activity, and MMP reduction were significantly diminished by SV.

**Conclusion:**

Data from this study suggest that SV can inhibit Al-induced cell death and apoptosis of PC12 cells via ameliorating OS.

## Introduction

Aluminum (Al), a non-essential and toxic element of high abundance in earth’s crust, is of widespread use in daily human life. Al is found in foods, drinking water, drugs, and cosmetic products and is absorbed by the human body mainly through gastrointestinal and respiratory systems. There is a wealth of experimental documents demonstrating the deleterious health effects of Al on the biological functions of most organs, notably the central nervous system (1). Disturbances in the synthesis of neurotransmitters and synaptic transmission (2), impairment in the activity of calcium channel and calcium signaling (3), alterations in post-translational modification and degradation of proteins (4, 5), changes in the expression of genes(6, 7), and induction of oxidative stress (OS) (8) are among the mechanisms proposed for Al neurotoxicity. In the case of OS, Al administration not only enhances the rate of reactions that generate reactive oxygen species (ROS) but also decreases the ability of non-enzymatic and enzymatic antioxidants in neutralizing ROS (9). Elevation of ROS has several adverse consequences for neurons, with neuronal death, most probably due to induction of apoptosis, among the most important ones (10). The apoptotic effects of Al in the nervous system due to its oxidative effects have been shown in many studies (9). Due to this mechanism of action of Al, the protective actions of several compounds with antioxidant activities on Al-induced apoptosis have been reported by several investigators (9, 11).

Sodium valproate (SV) is a histone deacetylase (HDAC) inhibitor used for the stabilization of mood in psychiatric patients(12). Its therapeutic effects have also been demonstrated in the combination therapy of cancers (13, 14). SV can control the transcription of many genes, including those involved in the control of OS upon inhibition of HDACs. The effect of HDACs inhibition in up-regulation of catalase (CAT) gene transcription in AML-2/DX100 cells is an example of such mechanism (15, 16). In addition, the therapeutic efficacy of valproate, as an antioxidant, in protecting the retina against oxidative damage resulting from ischemia-reperfusion has been demonstrated to be through the expression of antioxidant enzymes (17). 

 To the best of our knowledge, no study has evaluated the protective role of SV against Al-induced apoptosis. Therefore, the present study was conducted to explore the possibility of ameliorating Al-induced apoptosis by SV. To this end, the effects of aluminum maltolate (Almal) on cell death, apoptosis, and OS markers including ROS generation, mitochondrial membrane potential (MMP), and CAT activity were compared in the presence and absence of SV. 

## Materials and Methods

***Cell culture conditions and drug solution preparations***

The PC12 cell line was purchased from an Iranian cell bank (Pasture Institute, Iran). The cells were maintained in Dulbecco’s Modified Eagle’s Medium (DMEM, Gibco) supplemented with horse serum (10%), fetal bovine serum (5%) (Sigma-Aldrich), and penicillin-streptomycin (1%) (Sigma-Aldrich) at an atmosphere with 5% CO_2_ and 37 ^°^C. Aluminum chloride hexahydrate(Sigma-Aldrich) and 3-hydroxy-2-methyl-4-pyrone(Maltol) (Sigma-Aldrich) were used to prepare 25 mM stock solution of Almal as fully described in our previous investigation (18). The stock solution of SV (Sigma-Aldrich) was prepared in dimethyl sulfoxide (DMSO, Sigma-Aldrich). DMSO concentration was finally 0.1% in the culture medium of all experimental as well as control cells, which showed no significant toxic effects on viability of the cells.

**MTT test**

The effect of chemicals on the viability of PC12 cells was evaluated using 3-(4,5-dimethylthiazol-2-yl)-2, 5-diphenyltetrazolium bromide (MTT) test. Briefly, 24 hr after culturing PC12 cells in 96 well plates (5×10^3^/well) they were treated with different concentrations of Almal (100–2000 µM) and SV (50–1500 µM). Forty-eight hours after the treatments, MTT assay was done upon addition of MTT solution to the culture medium. The formed formazan was then solubilized by DMSO, and the absorbance of the colored solution was measured using a plate reader at a wavelength of 490 nm (18).

***Apoptosis assay using Annexin V/7-AAD flow cytometric method***

To assess the possible protective effects of SV against Almal-induced apoptosis, flow cytometric analyses were performed using Annexin V/7ADD (Apoptosis Detection kit I; BD Pharmingen) staining method. In brief, PC12 cells were cultured in T_25_ culture flasks and exposed to 1000 µM of Almal without or with the presence of SV (50, 100, 200, and 400 μM) for 48 hr. After washing the treated cells in cold phosphate-buffered saline (PBS), they were suspended in 1 ml of ice-cold binding buffer and stained with Annexin V/7-AAD at room temperature in darkness. Apoptosis was detected using a FACS Calibur flow cytometer (BD Bioscience, USA). The percentage of total apoptosis (early + late apoptotic cells) was calculated for each sample. 

***Flow cytometric assay of ROS***

The level of ROS in treated and control cells was measured through a flow cytometric method using DCFDA (Sigma). In this assay, DCFDA is oxidized in the presence of ROS and becomes DCF green fluorescent. Briefly, PC12 cells were concomitantly incubated with Almal (1000 µM) and SV (50, 100, 200, and 400 μM). Tert-butyl hydroperoxide (TBHP, 100 µM) was used as a positive control. After treatment, suspension of PC12 cells (150 µl) was washed in PBS and stained for 30 min with DCFDA (20 μM in Ringer buffered solution) at 37 °C in darkness. DCFDA-stained PC12 cells were washed and re-suspended in PBS. The stained cells were then excited at 488 nm, and the emitted fluorescent light (520 nm) was measured in the FL-1 channel of a FACS Calibur (BD). 

***Measurement of mitochondrial membrane potential***

Rhodamine 123 (Rh123) fluorescent dye was used to assess the alteration in the MMP of PC12 cells following treatment with Almal. Rh123 is absorbed and accumulated in the mitochondria quantitatively and in direct proportion to MMP. Any dissipation in MMP caused a decrease in mitochondrial accumulation and a decrease in fluorescent intensity of Rh123 (19). Briefly, 4 × 10^5^ PC12 cells were plated for 24 hr. The cells were then concomitantly exposed to Almal (1000 μM) and SV (50, 100, 200, and 400 μM) for 48 hr. After the treatment, the cells were washed twice with PBS and stained with Rh 123(1 μM) for 30 min in darkness. After washing the cells in PBS, they were excited at 490 nm, and the emitted fluorescence light at 520 nm was measured.

***CAT activity assay***

Due to the close relationship between CAT activity and Al-induced OS and sensitivity and availability of the method, we measured CAT activity to assess the cell response to OS. In brief, after treating PC12 cells with Almal (1000 μM) and SV (50, 100, 200, and 400 μM) for 48 hr, the cells were lysed upon sonication on ice and cell lysates were centrifuged (13000 g at 4^ °^C for 20 min) to separate the supernatant solutions. CAT activity of the supernatant solution was assayed based on the decomposition rate of H_2_O_2_ at 25 ^°^C, which caused a reduction in absorbance of the solution at 240 nm (4). Bradford method was used for determination of protein concentration in the supernatant. The activity of CAT (units/μg protein) in each experimental group was calculated and reported as a percentage of the enzyme activity in the control PC12 cells.

***Statistical analyses***

 SPSS statistical program (version 15) was used to analyze the data. The mean and standard deviations of each of the parameters (obtained from at least three independent experiments) were calculated. The presence of significant differences (*P*-value<0.05) between the means of the groups were evaluated using one-way analysis of variance (ANOVA). LSD *post hoc* test was then used for multiple comparisons. The IC_50_ values for Almal in MTT assay experiments were calculated using the GraphPad Prism 5 software (ver 5.01).

## Results

***The effects of Almal and SV on cell viability***

**Figure 1:**
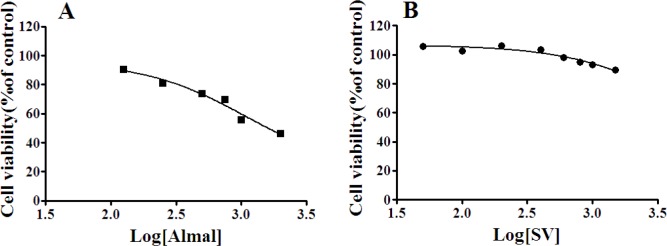
The effects of Almal (A) and SV (B) on cell viability. Forty-eight hours after treatment of PC12 with Almal (0–2000 µM) or SV (0–1500 µM) the cell survival was determined using the MTT method. GraphPad Prism software was used for analysis of the data and calculation of IC_50_ values

SV: Sodium valproate; MTT: 3-(4,5-dimethylthiazol-2-yl)-2,5-diphenyltetrazolium bromide

In this *in vitro* study, we firstly tested different doses of Almal and SV against PC12 cell survival (Figure 1). As seen, Almal decreased the viability of PC12 cells (Figure 1A) dose-dependently and significantly (IC_50_ =1096 µM). However, SV did not change the viability of PC12 cells at concentrations ranging from 50 up to 1000 µM (Figure 1B). However, higher concentrations of SV decreased the viability of PC12 cells significantly. Based on the above results, 1000 µM of Almal and 50–400 µM of SV were used for further experiments.

***The effects of SV on Almal-induced PC12 cell death***

**Figure 2:**
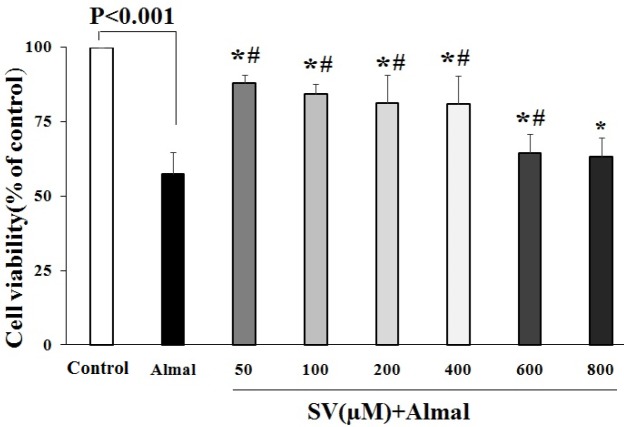
The effects of SV on cell death induced by Almal. MTT results revealed the protective effects of SV (50–600 μM) against Almal (1000 µM)-induced cell death. The data obtained from five independent MTT tests were analyzed using one-way ANOVA followed by the LSD *post hoc* test. The histogram represents mean±SD of viable cells. * and # show significant differences at *P*-value<0.05 compared with control and Almal-treated groups, respectively

SV: Sodium valproate; MTT: 3-(4,5-dimethylthiazol-2-yl)-2,5-diphenyltetrazolium bromide

Treatment of PC12 cells with SV at a concentration ranging from 50 up to 600 μM diminished Almal-induced cell death and significantly increased the viability of PC12 cells compared with the cells treated with Almal alone (Figure 2). The highest protective effects were obtained at 50 μM concentration of SV. At higher concentrations, the protective effects of SV were decreased, and at 800 μM concentration, SV showed no protective effects against Almal-induced cell toxicity. 

***The effect of SV on apoptosis induced by Almal***

**Figure 3:**
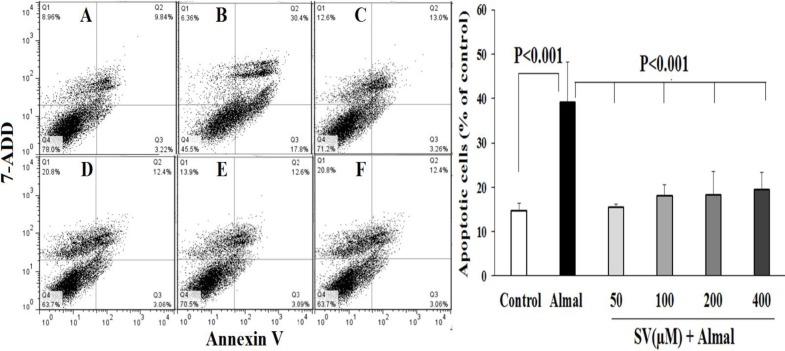
The ameliorating effect of SV on the apoptosis of PC12 cells induced by Almal. The flow cytometric graphs illustrate the percentage of viable cells (Q4), apoptotic cells (early: Q3 + late: Q2), and dead cells (Q1) in the control group (A) and the PC12 cells treated with 1000 µM of Almal alone (B) or Almal (1000 µM) in the presence of 50 µM (C), 100 µM (D), 200 µM (E), and 400 µM of SV (F). The histogram reveals the mean±SD of total apoptotic cells in various experimental groups. One-way ANOVA and LSD *post hoc *test were used for analysis of the data

Figure 3 demonstrates the effects of SV on Almal-induced apoptosis. The data reveals that Almal (1000 µM) increases the percentage of total apoptotic cells (Q2+Q3) and reduces the viable cells compared with the control group (*P*-value<0.001). Concomitant use of SV (50–400 μM) and Almal, diminished the percentage of apoptotic cells and increased cell viability as compared with the cells that were exposed to Almal alone (*P*-value<0.001). 

***The protective effects of SV on Almal-induced ROS generation in PC12 cells***

**Figure 4:**
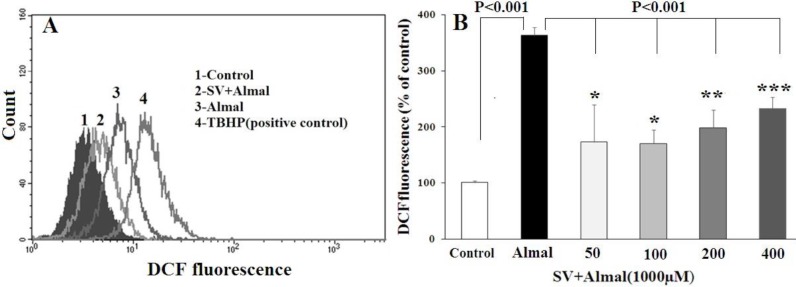
Flow cytometric analysis of intracellular ROS content in the PC12 cells treated with Almal and varying concentrations of SV. A: the representative spectra of fluorescent DCF in the cells treated with Almal, Almal+SV, and TBHP as a positive control. B: the comparative analysis of DCF fluorescence in various experimental groups. Each histogram represents mean ± SD of at least three ROS determination experiments. *, **, and *** show significant difference compared with the control cells at* P*-value<0.05, *P*-value< 0.01, and *P*-value<0.001, respectively

SV: Sodium valproate; ROS: Reactive oxygen species

Figure 4 shows the results obtained from ROS determination in PC12 cells using DCF flow cytometric assay. A significantly higher level of ROS (*P*-value< 0.001) was detected in the PC12 cells treated with Almal (1000 μM) compared with the control group. A decrease in the ROS content was detected in the cells exposed concomitantly with Almal and various concentrations of SV (50, 100, 200, and 400 μM) as compared with the cells exposed to Almal alone (*P*-value<0.001). A significant difference was also observed between the ROS content of the cells treated with Almal+SV compared with untreated control cells. However, no significant difference was detected in ROS content between cells treated with various concentrations of SV.

***Effect of Almal and SV on the mitochondrial membrane potential***

**Figure 5:**
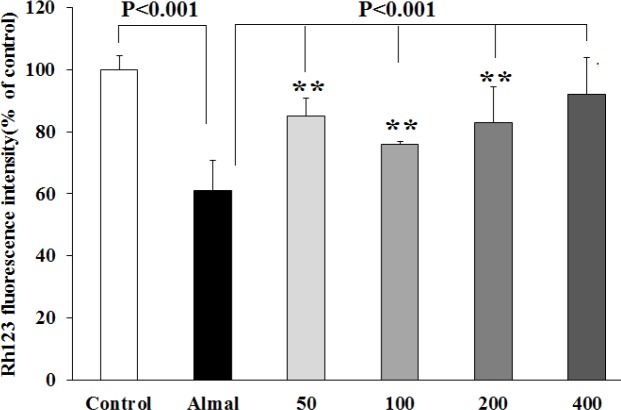
Evaluation of mitochondrial membrane potential of the PC12 cells using Rh123 fluorescence. The histograms represent mean±SD values of Rh123 fluorescence obtained from the PC12 cells co-treated with Almal (1000 μM) and SV (50–400 μM). ** shows significant difference at *P*-value<0.01 compared with the control untreated cells

The effects of Almal and SV on the MMP levels were quantified, using Rh123 fluorescence. As depicted in Figure 5, PC12 cells exposed to Almal (1000 µM) demonstrated a 40% decrease in MMP compared with the control untreated cells (*P*-value< 0.001), indicating dissipation of MMP by Almal. Co-treatment with SV significantly restored MMP by a decrease of 24%, 16%, 23%, and 30% of the control at 50, 100, 200, and 400 μM concentrations, respectively (*P*-value<0.001), suggesting the role of SV in recovering the mitochondrial membrane depolarization induced by Almal (Figure 5).

***Effect of Almal and SV on catalase activity***

**Figure 6:**
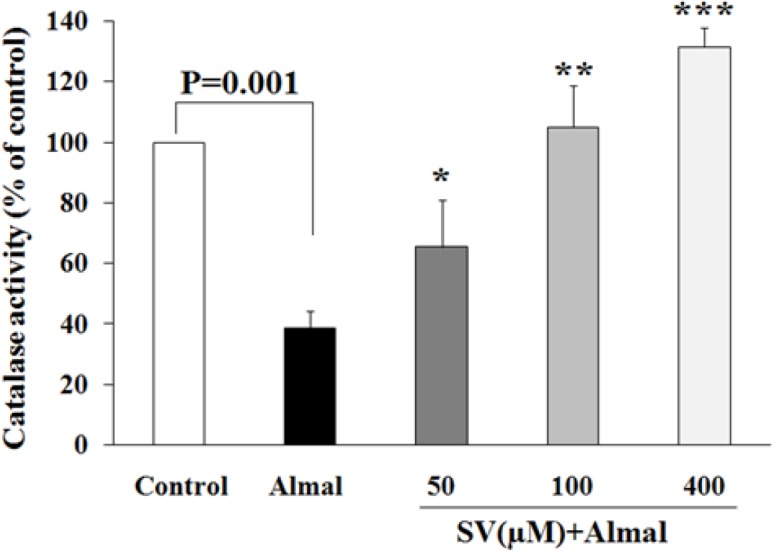
Evaluation of CAT activity of PC12 cells co-treated with Almal (1000 µM) and SV (50–400 μM). The histograms represent mean±SD of CAT activity obtained from at least three experiments. One-way ANOVA and LSD *post hoc* test were used for the analysis of data. * shows significant difference at *P*-value<0.05 compared with the control group, ** and *** show significant difference at* P*-value<0.01 and* P*-value<0.001 compared with the Almal group, respectively

SV: Sodium valproate; CAT: Catalase activity

CAT is among the major enzymes in cellular defense against OS. Figure 6 demonstrates the effects of Almal on CAT activity, together with the protective effects of SV. As shown, Almal reduced the activity of CAT to about 40% of the control cells (*P*-value<0.001). Co-treatment with Almal and SV (50, 100, and 400 μM) dose-dependently and significantly restored the activity of CAT compared with the cells treated with Almal alone.

## Discussion

Association of Al-induced OS with the pathogenesis of neurodegenerative disease such as amyotrophic lateral sclerosis,Parkinson’s disease, and Alzheimer’s disease has been indicated by numerous investigators (20, 21). Recent investigations have also revealed the protective effects of SV as an antioxidant against several neurotoxic compounds (22, 23). Our results demonstrated that SV at concentrations with no toxic effect on cell viability (50–400 µM) could inhibit Al-induced cell death and apoptosis. Moreover, SV-treatment significantly suppressed the effects of Almal on ROS generation, MMP loss, and CAT activity. Collectively, the observed protective effects of SV in the present and previous studies suggest that SV might be considered as a new modality for the prevention of neurodegeneration.

The findings of apoptosis assay clearly showed that SV diminishes the Almal-induced apoptosis of the PC12 cells. The protective role of SV against neuronal cell apoptosis has also been indicated in several previous studies. In the primary culture of neuron-glia cells obtained from rat midbrain, it has been demonstrated that valproate can protect the neurons against neurotoxicity induced by lipopolysaccharides (24). Recently, Zhang *et al.* have shown that SV can protect SH-SY5Y cells against MPP^+^-mediated apoptosis (23). Furthermore, it has been found that treatment with valproate protects the neural progenitor cells against H_2_O_2_-induced cell death through modulation in the expression of anti-apoptotic and pro-apoptotic proteins (25). In addition to neurons, the protective effects of SV have been revealed in other cells. In H9c2 cardiomyoblast cells, valproate rescued the cells from COCl2-induced hypoxia through up-regulating the expression of Bcl-2 (26). However, in ARPE-19 cells, a human retinal epithelial cell line, valproate had no significant protective effect against OS-induced apoptosis (32), indicating the importance of the cell type and the stimulator upon the response of cells to the protective effects of valproate. 

Several mechanisms including induction of autophagy (23, 27), inhibition of mitochondrial-mediated apoptosis (23, 28), inhibition of ROS generation (29), and induction of the expression of antioxidant enzymes are suggested for the anti-apoptotic effects of valproate (30). One possible mechanism that may describe the observed anti-apoptotic effects of SV and is supported by the findings of our study is the inhibition of Al-induced ROS generation by SV. Results of ROS determination and also of CAT activity assays revealed that SV diminishes the Al-induced ROS generation and increases the CAT activity. Similar findings have been reported by Kabel *et al.* (31) on the effects of valproate on bleomycin-induced ROS generation in rats in which valproate treatment decreased OS through increases in the activities of CAT and superoxide dismutase. Moreover, in the rat retina (17) and in retinal pigment epithelial cells (32), the effective role of valproate in increasing CAT activity through enhancement of the expression of CAT and other antioxidant enzymes genes has been demonstrated. 

Mitochondria are the key organelles for cellular energy production, especially for the neurons with an abundant demand for energy for their physiological functions. Therefore, mitochondrial dysfunction can lead to neuronal damage. Indeed, an association between mitochondrial dysfunction and neurodegenerative diseases has been demonstrated (33). MMP supplies the energy source for the synthesis of ATP. Therefore, any dissipation or loss of MMP can cause a failure in the generation of ATP. Furthermore, loss of MMP leads to excessive production of ROS and release of cytochrome C to the cytoplasm, which may ultimately trigger mitochondrial-mediated apoptosis (34). The effect of Al in dissipation of MMP has been indicated previously. In the present study, we found a 40% reduction in the MMP level of PC12 cells following exposure to Almal, whereas treatment with SV restored the MMP approximately similar to that of the control cells. Consistent with our findings in the current study, previous studies have reported that valproate diminished the MMP loss in the spinal trigeminal neurons following treatment with nitroglycerine (35) and also in SH-SY5Y cells following exposure to MPP+ (23), suggesting the potential protective effects of valproate against neurotoxin-mediated mitochondrial damage.

SV: Sodium valproate

SV: Sodium valproate

## Conclusion

Findings from this study provide evidence on the antioxidant potential and the protective effects of SV against Almal-induced ROS generation, MMP dissipation, and apoptosis in PC12 cells. Therefore, SV and probably other HDACs can be considered as new modalities in the therapeutic management of neurodegenerative diseases. Further investigations are recommended to shed light on molecular events involved in the protective effect of SV against Al toxicity.
